# Targeting the neurovascular unit in retinal fibrosis: mechanisms and therapeutic perspectives

**DOI:** 10.3389/fmed.2026.1776869

**Published:** 2026-03-05

**Authors:** Wenyang Xu, Jie Zhang, Yanyu Shangguan, Ruoning Luo, Yanming Zhu, Yanlong Bi, Li Chen, Bing Li

**Affiliations:** 1 Department of Ophthalmology, Tongji Hospital, School of Medicine, Tongji University, Shanghai, China; 2 Department of Ophthalmology, Yangpu Hospital, School of Medicine, Tongji University, Shanghai, China

**Keywords:** epithelial-mesenchymal transition, Jagged/Notch signaling pathway, myofibroblast, neurovascular unit, retinal fibrosis, TGF-β signaling pathway, therapeutic targets

## Abstract

Retinal fibrosis, a severe complication observed in conditions like age-related macular degeneration and diabetic retinopathy, characterized by aberrant myofibroblast activation and excessive extracellular matrix (ECM) deposition, which ultimately led to irreversible visual impairment. Currently, the mechanisms underlying retinal fibrosis remain unclear and existing treatments remain incompletely understood. Thus, a comprehensive understanding of disease mechanisms, together with the development of innovative therapeutic approaches, is essential for advancing effective treatment strategies. This review systematically examines the pathogenesis of retinal fibrosis from the perspective of the neurovascular unit (NVU), with a particular focus on the roles of endothelial cells, pericytes, and glial cells in fibrotic processes. It highlights key fibrotic mechanisms, including epithelial mesenchymal transition (EMT) as well as macrophage and pericyte-to-myofibroblast transitions (MMT/PMT). It further analyzes the molecular mechanisms that regulate myofibroblast activation and extracellular matrix deposition. Additionally, this review outlines potential therapeutic targets for the treatment of retinal fibrosis.

## 1 Introduction

Fibrotic diseases arise from the excessive accumulation of extracellular matrix components, leading to structural tissue damage and loss of organ function (1). Retinal fibrosis is primarily driven by the trans-differentiation of mesenchymal cells into myofibroblasts, which promote fibro-cellular proliferation and ultimately lead to irreversible vision loss (2). These transitions reflected in endothelial cells, pericytes, microglia, macrophages, and other cellular sources (3).

**Figure 1:**
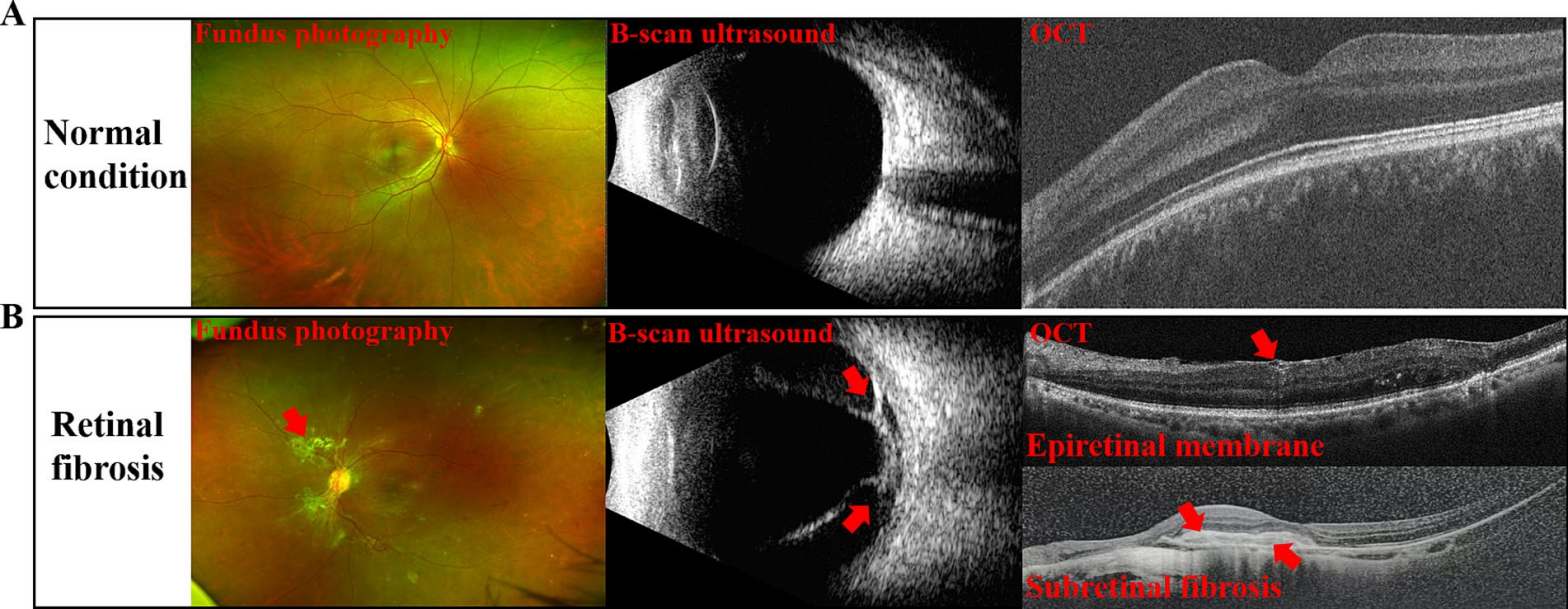
Imaging findings of retinal fibrosis. **(A)** Fundus photographs, B-scan ultrasound and OCT images of normal subjects; **(B)** Fundus photographs, B-scan ultrasound images and OCT of patients with retinal fibrosis (patients with the proliferative form of diabetic retinopathy, PDR), with the fibrotic areas indicated by red arrows.

Retinal fibrosis manifests as a complication in retinopathy such as age-related macular degeneration (AMD) and diabetic retinopathy (4) (Table 1). However, there are no comprehensive treatment protocols or approved drugs currently available for advanced stages of retinal fibrosis. Thus, it is critical to explore the underlying mechanisms and identify effective therapeutic strategies (Figure 1).

**Table 1:** Specific characteristics of retinal fibrosis in different diseases.

Disease	Primary anatomical site of fibrosis	Main cell populations driving fibrosis	Primary cellular transition process
Neovascular Age-Related Macular Degeneration (nAMD) (63)	Subretinal interface (choroidal neovascularization accompanied by subretinal fibrosis)	Retinal pigment epithelial cells, choroidal endothelial cells	Epithelial-mesenchymal transition (EMT) of RPE; Endothelial-mesenchymal transition (EndMT) of choroidal endothelial cells
Proliferative Diabetic Retinopathy (PDR) (64, 65)	Preretinal interface (fibrovascular membranes),	Endothelial cells, pericytes, glial cells	Endothelial-mesenchymal transition (EndMT)
Proliferative Vitreoretinopathy (PVR) (66)	Vitreoretinal interface (epiretinal membranes, subretinal membranes, vitreous membranes)	Retinal pigment epithelial cells, glial cells, macrophages	Wound healing-like epithelial-mesenchymal transition (EMT) of RPE; Glial cell activation
Idiopathic Epiretinal Membrane (iERM) (67)	Preretinal interface (inner surface of the macula)	Müller glial cells	Glial-to-mesenchymal transition (GMT) of Müller cells

Emerging evidence indicates that retinal fibrosis arises as a downstream consequence of chronic inflammation and pathological neovascularization, serving as a final common pathway in multiple advanced fundus diseases. Retinal pigment epithelial cells are also a principal source of myofibroblasts in subretinal fibrosis, undergoing epithelial mesenchymal transition in response to stimuli such as TGF-β, which confers migratory capacity and enhanced extracellular matrix production. Choroid-derived fibroblasts and pericytes can be directly activated by inflammatory mediators, resulting in cellular proliferation and excessive extracellular matrix deposition that contributes to fibrotic scar formation. Meanwhile, infiltrating monocytes from the choroid differentiate into macrophages. Through paracrine release of profibrotic mediators such as TGF-β, IL-6, and Galectin-3, these macrophages drive cellular activation and trans-differentiation, functioning as a key signaling hub in retinal fibrosis. The Neurovascular unit (NVU), a foundational concept in vascular and neurological pathology, has emerged as an important target in retinal fibrosis research. The NVU framework first underscores the symbiotic and highly coordinated interactions between neural cells and the vascular system (5). The coordinated interactions among these NVU components dynamically shape the blood-retinal barrier (BRB), a structure essential for preserving retinal homeostasis and functional stability (4).

This review examines the pathogenesis of retinal fibrosis and identifies potential therapeutic targets from NVU centered perspective, with the goal of informing the development of effective antifibrotic therapies.

## 2 NVU and retinal fibrosis

Myofibroblasts in retinal fibrosis originate from multiple cellular sources. Beyond retinal pigment epithelial cells undergoing epithelial mesenchymal transition, Müller cells, pericytes, choroidal fibroblasts, and infiltrating monocyte-derived macrophages can differentiate into myofibroblast-like cells within the pathological microenvironment, exhibiting excessive extracellular matrix production (6, 7). Through gap junction communication, receptor-dependent signaling, and paracrine cytokine release, the NVU establishes a bidirectional regulatory loop with myofibroblasts. This interaction amplifies profibrotic activation within the retinal microenvironment, coupling inflammatory stimuli to progressive extracellular matrix deposition. Accordingly, the neurovascular unit functions both as the shared structural niche for fibrogenic effector cells and as a central platform for integrating and amplifying profibrotic signaling.

Current evidence indicates that various cell types in retina can trans-differentiate into myofibroblasts in retinal fibrosis through processes including epithelial-to-mesenchymal transition (EMT), macrophage and pericyte-to-myofibroblast transitions (MMT/PMT) (8, 9). Additionally, pathological conditions such as inflammation or hemorrhage, can activate glial cells secrete pro-inflammatory cytokines and growth factors like vascular endothelial growth factor (VEGF) and transforming growth factor-beta (TGF-β), which promote myofibroblast proliferation and accelerate fibrotic progression (10, 11). This pathological process arises from complex and coordinated interactions among multiple cell types within the NVU.

Endothelial cells, as the core component of the NVU, directly contact blood and establish the vascular barrier via tight junctions (12). During endothelial-mesenchymal transition (End-MT) in fibrotic matrix production, endothelial markers such as cluster of differentiation 31 (CD31) and VE-cadherin were downregulated and mesenchymal markers were acquired (2), a process crucial for fibroblast activation.

Pericytes are phagocytic cells embedded within capillary basement membranes (13). Pericytes interact with endothelial cells to maintain blood flow stability and play a role in angiogenesis. Additionally, they may serve as a source of myofibroblasts, for TGF-β1 treatment inducing PMT. Pericyte detachment increases microvascular permeability and structural degeneration, eventually contributing to fibrosis (14).

Astrocytes, primarily located in the nerve fiber and inner nuclear layers (15), are essential for retinal vascular development. Astrocytes also participate in inflammatory responses by presenting antigens, phagocytosing pathogens, and secreting cytokines and chemokines such as tumor necrosis factor-alpha (TNF-α), interleukin-1 beta (IL-1β) and matrix metalloproteinase-2 (MMP-2) (16). Retinal astrocytes can also act as structural scaffolds that facilitate fibroblast differentiation and promote collagen synthesis (17).

Müller cells, the radially oriented glial cells that span the full thickness of the retina (15), form synaptic interactions with neurons and provide essential metabolic and functional support (18). They become activated in response to mechanical stress, retinal injury, ischemia, hyperglycemia, or inflammatory cytokines. Mechanical traction generated by epiretinal membranes induces reactive gliosis in Müller cells, thereby driving myo-fibroblastic trans-differentiation and contributing to fibrotic scar formation (19).

**Figure 2:**
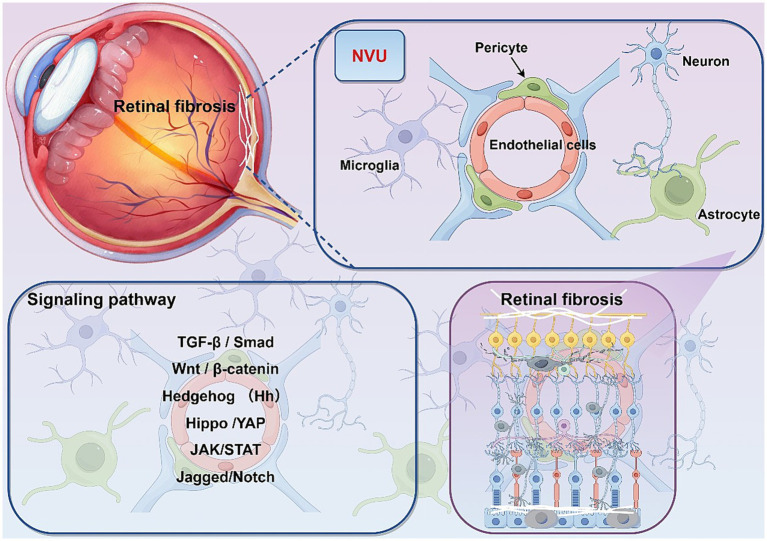
Analysis of the composition of the retinal neurovascular unit and the pathways involved in retinal fibrosis.

Microglia, specialized glial macrophages residing near retinal vasculature, modulate neuronal survival and inflammation through the secretion of trophic factors, antioxidants, and cytokines (18). In epiretinal membranes (ERMs), these microglia release TGF-β1, promoting the trans-differentiation of epithelial cells into myofibroblast-like cells, thereby linking neuroinflammation to fibrosis (20) (Figure 2).

## 3 Fibrosis-associated signaling pathways

**Figure 3:**
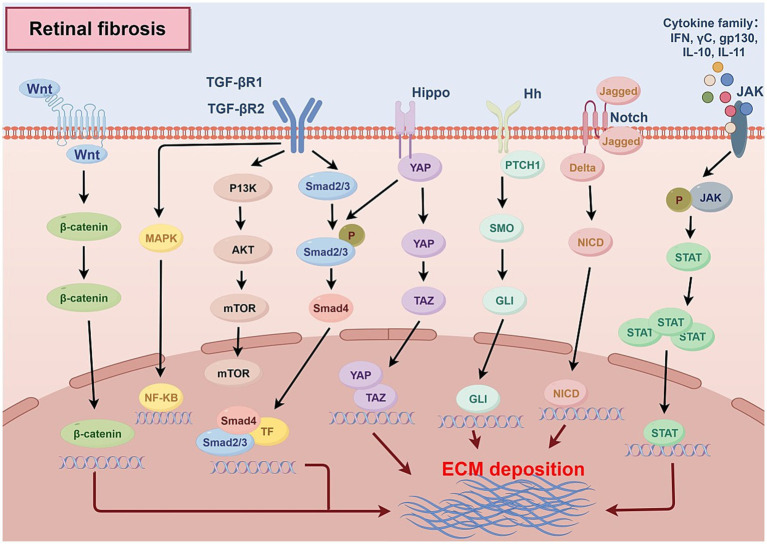
Fibrosis-associated signaling pathways. Hh, Hedgehog; MAPK, Mitogen-activated protein kinase; TF, Transcription Factor; YAP, Yes-associated protein; TAZ, Transcriptional coactivator with PDZ-binding motif; NF-KB, Nuclear Factor kappa-light-chain-enhancer of activated B cells; SMO, Smoothened; NICD, Notch Intracellular Domain; JAK, Janus kinase; STAT, Signal Transducer and Activator of Transcription.

Multiple intracellular signaling pathways within NVU cells contribute to the pathogenesis of retinal fibrosis (Figure 3).

### 3.1 TGF-β signaling pathway

TGF-β is the most well-established mediator of EMT and fibrosis. As a multifunctional cytokine, TGF-β plays a crucial role in regulating tissue homeostasis and repair, immune and inflammatory responses, extracellular matrix (ECM) deposition, and cell differentiation and growth. The TGF-β signaling pathway is initiated by a low-affinity heteromeric receptor complex composed of TβRI and TβRII, which activates distinct downstream signaling cascades to regulate gene transcription. These signaling pathways are generally classified into SMAD-dependent and non-SMAD-dependent pathways (21).

Although all three isoforms of TGF-β are expressed in fibrotic tissues, tissue fibrosis predominantly results from the activity of TGF-β1 (21). Under pathological conditions, TGF-β1 overexpression promotes EMT and excessive ECM deposition, driving fibrotic progression by activating fibroblasts into myofibroblasts and inducing pericyte trans-differentiation into myofibroblasts (3).

#### 3.1.1 The classical Smad-dependent pathway

In the canonical pathway, TGF-β binds to a heterodimeric receptor complex composed of TGF-β receptor type I (TβRI) and TGF-β receptor type II (TβRII). Ligand binding activates TβRI, which subsequently recruits and phosphorylates Smad2/3. The phosphorylated Smad2/3 then associates with Smad4 to form a trimeric complex that trans-locates into the nucleus, where it regulates the transcription of mesenchymal genes (3).

Smad proteins are key intracellular mediators of TGF-β signaling and are indispensable for relaying signals from cell-surface receptors to the nucleus. In the retina, TGF-β1 has been shown to induce the nuclear translocation of Smad2/3, thereby promoting a fibrotic phenotype and enhancing extracellular matrix production in human retinal pigment epithelial cells. Importantly, ocular fibrosis does not occur in isolation; it is frequently accompanied by disruption and aberrant remodeling of NVU (22). Activated myofibroblasts secrete abundant collagen and other ECM components, reshaping the native mechanical and biochemical microenvironment of the tissue (23). Such aberrant remodeling of the ECM disrupts the normal spatial organization and signaling interactions among neurons, glial cells, and blood vessels.

In addition, the negative feedback regulation of the pathway constitutes an intrinsic antifibrotic barrier within the NVU. For example, overexpression of Smad7 in fibroblasts effectively antagonizes TGF-β2 induced type I collagen synthesis and Smad2 phosphorylation (3). These findings suggest that targeting TGF-β represents a potential strategy to restore the balance between profibrotic and antifibrotic signaling and to inhibit aberrant remodeling of the NVU.

#### 3.1.2 Non-classical TGF-β pathways

All TGF-β-activated pathways and downstream cascades involving post-translational modifications such as phosphorylation, acetylation, sumoylation, ubiquitination, and protein–protein interactions are collectively referred to as non-SMAD signaling pathways. These pathways include Phosphatidylinositol 3-kinase/Protein Kinase B pathway (PI3K/Akt), mitogen-activated protein kinase (MAPK), and the Jagged/Notch signaling pathway. At the retinal level, TGF-β can directly induce EMT in retinal pigment epithelial (RPE) cells via Notch signaling, conferring enhanced migratory and proliferative capacities as well as robust ECM secretion, which promotes the progression of subretinal fibrosis (24).

In addition, TGF-β2 can drive Endo-MT in vascular endothelial cells via YAP signaling, leading to loss of barrier function and acquisition of mesenchymal characteristics, thereby directly contributing to retinal fibrotic scar formation (25).

Through these pathways, TGF-β promotes aberrant cellular phenotypic transitions and pathological ECM deposition, fundamentally altering the microenvironment of the NVU, thereby influencing the retinal fibrotic process (26).

### 3.2 Wnt signaling pathway

Wnt signaling, a highly conserved pathway, plays a pivotal role in development of fibrosis. Research suggests that Wnt signaling is involved in cellular senescence, the progression of age-related diseases, and the fibrotic response to tissue injury (27). Dysregulated Wnt signaling is closely associated with myofibroblast activation, EMT and fibrosis (3).

The Wnt signaling pathway is primarily divided into two categories: the canonical pathway, involving β-catenin, and the non-canonical pathway, which is β-catenin-independent. Activation of the canonical Wnt pathway can drive EMT in RPE cells within the NVU and may also influence the activation status of glial cells, such as Müller cells (28). Activation of the non-canonical Wnt pathway forms a networked interaction with the TGF-β signaling pathway, regulating the fibrotic process. Collectively, these pathways form a synergistic network that amplifies profibrotic signaling and drives pathological remodeling of the NVU (29).

Current evidence suggests that activation of the canonical Wnt/β-catenin pathway is linked to retinal fibrosis. In AMD, the classical Wnt/β-catenin pathway driven EMT in retinal cells facilitates subretinal fibrosis (30). In proliferative vitreoretinopathy (PVR), canonical Wnt/β-catenin signaling drives EMT in core NVU cells, namely RPE cells, triggering a cascade of events that ultimately result in fibrous membrane formation and disruption of retinal architecture (31).

### 3.3 Hedgehog (Hh) signaling pathway

The Hh protein is a signaling molecule secreted by epithelial cells that regulates cell proliferation, differentiation, morphology, and tissue remodeling through autocrine and paracrine signaling, especially during regeneration following injury (32). Studies have shown that high-glucose conditions upregulate Hh protein expression in human retinal microvascular endothelial cells, and activation of this pathway markedly promotes endothelial cell proliferation and migration. This process may be mediated by the regulation of PLCγ1, Akt, and Erk phosphorylation downstream of G protein-coupled receptor (GPCR) signaling, thereby directly affecting the functional integrity of the BRB. In addition, Hh signaling can activate RPE cells and other retinal cells, which constitute the primary cellular sources of subretinal fibrotic and proliferative membranes.

Ocular fibrosis is frequently accompanied by pathological neovascularization, and Hh signaling has been shown to be activated in animal models of both choroidal and retinal neovascularization. Activated Hh signaling promotes angiogenesis and couples vascular growth to extracellular matrix deposition by upregulating profibrotic factors such as VEGF and α-smooth muscle actin (α-SMA), collectively driving disordered remodeling of the NVU (33).

As a key paracrine signal, Hh signaling regulates communication among multiple cell types within the NVU, including endothelial cells, pericytes, RPE cells, and glial cells. Disrupted intercellular crosstalk can induce EMT and Endo-MT, promote myofibroblast activation, and enhance excessive ECM deposition, ultimately contributing to the development of retinal fibrosis (34).

### 3.4 Hippo/YAP pathway

The Hippo pathway is an evolutionarily conserved signaling cascade that critically regulates organ development, maintains epithelial homeostasis, promotes tissue regeneration, facilitates wound healing and modulates immune responses. This pathway is mediated by the transcriptional coactivators YAP (Yes-associated protein) and TAZ (transcriptional coactivator with PDZ-binding motif) (35). Activation of the Hippo pathway suppresses YAP/TAZ activity through phosphorylation. Conversely, inactivation of the Hippo pathway leads to dephosphorylation of YAP/TAZ, enabling their translocation into the nucleus where they interact with TEAD1-4 transcription factors to promote gene expression (36). Dysregulation of Hippo signaling and abnormal YAP/TEAD activity are strongly associated with the development of fibrosis (35). These are the key mechanisms of the Hippo/YAP pathway in fibrosis.

YAP/TAZ, critical effectors of the Hippo pathway, promote fibrosis by regulating the expression of pro-fibrotic factors, which in turn drive ECM synthesis (37). For instance, activated YAP/TAZ can interact with TGF-β-activated SMAD transcription factors to facilitate myofibroblast trans-differentiation and enhance ECM production (38). YAP/TAZ can induce pro-fibrotic effects in response to matrix stiffness even in the absence of exogenous TGF-β. Studies suggest that elevated nuclear YAP/TAZ levels may orchestrate fibrogenic gene networks essential for transmitting and responding to microenvironmental signals (39).

Furthermore, the interaction between YAP/TAZ and TEAD can mediate EMT to drive fibrosis. In the kidney, this process induces EMT in renal tubular epithelial cells. Transformed cells overproduce extracellular matrix components, such as laminin and type IV collagen, thereby contributing to the development of renal fibrosis (40).

### 3.5 JAK/STAT pathway

The JAK/STAT signaling pathway is a cascade that modulates gene expression and plays a vital role in cellular processes including proliferation, differentiation, apoptosis, and autophagy (41).

In the retina, the JAK2/STAT3 pathway mediates Müller cell activation, a key feature of the retinal fibrotic response. Chronic low-grade inflammation provides a common substrate for fibrosis, and the JAK/STAT pathway—particularly STAT3—drives the differentiation of CD4^+^T cells into proinflammatory and profibrotic Th17 subsets, which secrete cytokines such as IL-17. These factors continuously act on endothelial cells, RPE cells, and glial cells within the NVU, disrupting immune homeostasis and creating a microenvironment conducive to fibrotic progression.

Moreover, the JAK/STAT pathway crosstalk with the core fibrotic TGF-β/Smad signaling cascade in Müller cells and RPE cells, forming a synergistic regulatory network that amplifies the transcriptional program of profibrotic genes and sustains the fibrotic response.

Therefore, the JAK/STAT signaling pathway promotes ocular fibrosis and pathological remodeling of the NVU by driving phenotypic transitions in structural NVU cells, shaping a chronically profibrotic inflammatory microenvironment, and integrating with core pathways such as TGF-β.

### 3.6 Jagged/Notch signaling pathway

The Notch signaling pathway is a highly conserved intercellular communication mechanism that plays essential roles in embryonic development and the maintenance of tissue homeostasis (3). In mammals, Notch activation is initiated by the binding of two classes of canonical ligands, Delta-like ligands and Jagged1/2, to their respective receptors. Following ligand-receptor binding, the Notch intracellular domain (NICD) is cleaved by γ-secretase and trans-locates to the nucleus, where it associates with the CSL family of transcription factors to induce target gene expression (42).

Activation of Jagged-1/Notch signaling promotes EMT in lens epithelial cells (LECs) following TGF-β2 stimulation, contributing to lens fibrosis in both *in vitro* and *in vivo* models. And blocking Notch signaling was shown to reverse these effects (43). Additionally, study revealed that activation of Notch signaling mediates Müller cell–driven retinal fibrosis, whereas inhibition of this pathway attenuates Müller cell gliosis and reduces ECM deposition, thereby slowing fibrotic progression (44). These findings offer foundational insights into the specific mechanisms of the Jagged/Notch pathway in retinal fibrosis and suggest potential therapeutic targets.

Overall, multiple signaling pathways interact to drive retinal fibrosis within the NVU, including TGF-β/Smad, Wnt/β-catenin, Hedgehog, Hippo/YAP, JAK/STAT, and Jagged/Notch. These pathways converge on common profibrotic mechanisms such as EndMT and EMT, myofibroblast activation, and excessive extracellular matrix deposition. Crosstalk among these cascades amplifies pathological remodeling of the NVU microenvironment, ultimately promoting structural disruption and fibrotic progression in retinal diseases.

## 4 Therapeutic targeting of fibrosis

Based on the pathways discussed above, the current therapeutic targets for fibrosis can be summarized as follows:

### 4.1 Myofibroblasts and the TGF-β pathway

Retinal pigment epithelial cells, Müller cells, and pericytes can become activated and transdifferentiate into fibroblast-like cells under pathological conditions, thereby further driving the fibrotic process. Consequently, targeting fibroblast activation and inhibiting their proliferation has become a major therapeutic focus in retinal fibrosis. Studies have demonstrated that inhibiting TGF-β-induced production of fibronectin and α-SMA in human fibroblasts, thereby modulating the activation and transition of myofibroblasts (45).

Various TGF-β-targeted therapies have been developed, including oligonucleotides, peptides, receptor blockers, antibodies, and small molecule inhibitors (46). For example, *nintedanib* and *pirfenidone* exert anti-fibrotic effects by inhibiting TGF-β signaling and reducing extracellular matrix gene expression (47, 48). Additionally, intravitreal administration of the herbal compound triptolide can diminish the extent of subretinal fibrosis by reversing TGF-β1/Smad-mediated EMT and EndMT (49). *Fenofibrate*, a selective PPARα agonist, significantly reduces subretinal fibrosis in VLDLR knockout mice by inhibiting TGF-β/Smad2/3 and Wnt/GSK3β signaling pathways (30). In recent years, emerging compounds such as *thrombospondin-1 (TSP-1)* and *biochanin-A (BCA)* have demonstrated promising potential as TGF-β-targeted therapies for fibrosis (50, 51).

However, despite their promising effects in experimental models, the clinical application of most TGF-β inhibitors for fibrosis remains challenging due to the complex nature of fibrotic processes and the essential physiological roles of TGF-β in the human body (26).

### 4.2 Other signaling pathways

In addition to the TGF-β pathway, other signaling pathways promote myofibroblast activation, recruitment, and ECM deposition either by inducing epithelial-mesenchymal transition (EMT) or through crosstalk with the TGF-β pathway. Thus, targeting these pathways has provided significant insights into potential therapeutic strategies for fibrosis.

For example, the Wnt/GSK-3β pathway can exert anti-fibrotic effects by synergistically inhibiting the TGF-β signaling pathway. Evidence indicates that glycogen synthase kinase 3β (GSK3β) inhibits epithelial-mesenchymal transition (EMT) in experimental proliferative vitreoretinopathy (PVR) by modulating both the Wnt/β-catenin and PI3K/Akt signaling pathways (52).

Furthermore, silencing the YAP gene within the Hippo pathway effectively inhibits TGF-β1-mediated gene activation (37). Additionally, selective activation of the Gα-coupled dopamine receptor D1 (DRD1) induces targeted inhibition of YAP/TAZ in mesenchymal cells, thereby modulating the fibrotic process (53). This highlights the therapeutic potential of selectively targeting YAP/TAZ activity in fibrotic diseases (35).

Recent research revealed that the Notch pathway interacts with the TGF-β pathway to play a pivotal role in Müller cell-driven retinal fibrosis. The administration of the γ-secretase inhibitor *RO4929097* in mouse models offers a promising therapeutic approach by targeting Notch signaling (44). Intravitreal injection of *RO4929097* inhibited both signaling pathways, decreased ECM protein overexpression, and prevented retinal fibrosis in the mouse model (44). Considering the pivotal role of Müller cells in retinal fibrosis and the encouraging results of this study, these findings have important implications for developing clinical therapies to prevent retinal fibrosis in humans.

Wnt, YAP/TAZ, and Notch signaling mediate the transition of retinal epithelial cells, Müller cells, and other NVU cells into myofibroblasts, thereby promoting the progression of retinal fibrosis. Thus, drug development targeting these pathways should be highlighted.

In Müller cell–driven models of retinal fibrosis, intravitreal RO4929097 inhibits TGF-β and Notch signaling and reduces subretinal fibrosis in mice, directly supporting a retina-specific rationale for clinical translation of Notch-targeted therapy. The Wnt pathway contributes to TGF-β–mediated fibrotic responses in retinal pigment epithelial cells and corneal fibroblasts; however, direct intervention studies targeting the retinal NVU remain limited. In contrast, the role of the Hedgehog pathway in ocular fibrosis has thus far been primarily elucidated in models of corneal, conjunctival, and orbital diseases, and its specific contribution to endogenous retinal fibrosis (e.g., proliferative vitreoretinopathy, subretinal fibrosis) still lacks systematic investigation. Therefore, translation of these pathways into antifibrotic therapies for retinal disease requires target validation and efficacy assessment in retina-specific models.

### 4.3 Targeting the complement system

Numerous complement proteins and regulators can be produced by various tissues, including the retina, which exhibits a greater capacity for regulating local complement activation (54).

Elevated plasma levels of C3a and C5a have been observed in patients with macular fibrosis associated with nAMD, highlighting their potential therapeutic role in retinal subretinal fibrosis (55). The complement system can promote fibrosis by sustaining inflammation, as complement mediators C3a and C5a recruit immune cells to sites of inflammation, amplifying inflammation and driving the progression of fibrosis (56).

Within the NVU, the complement system also promotes fibrosis by inducing MMT. Meanwhile, C5a promotes the production of pro-fibrotic factors such as TGF-β1 and TGF-β2 and mediates EMT through specific signaling pathways (57). Notably, C5 inhibition (BB5.1) has been shown to significantly reduce subretinal fibrosis (57).

Consequently, targeted therapeutic strategies aimed at the complement system, such as the use of C3 and C5 inhibitors, offer promising approaches to mitigate fibrosis by blocking their respective roles in this process (58).

Current evidence links the complement system to retinal fibrosis primarily through robust clinical associations, whereas mechanistic validation remains limited. Clinically, the extent of macular fibrosis in neovascular age-related macular degeneration correlates positively with plasma C3a and C5a levels, supporting a pathogenic role of complement activation. Experimentally, the anti-C5 monoclonal antibody BB5.1 significantly reduces subretinal fibrosis in a laser-induced choroidal neovascularization model, indicating the therapeutic potential of complement inhibition. Nevertheless, current mechanistic insights into complement-driven fibrosis, including its roles in macrophage-to-myofibroblast transition and epithelial mesenchymal transition, are primarily derived from systemic fibrosis models such as those of the kidney and lung. The downstream signaling pathways in retinal pigment epithelial and Müller cells remain incompletely characterized. Moreover, the complement system within the retina constitutes a locally synthesized and regulated network that is partially independent of systemic circulation. A central challenge for retinal-targeted complement therapies will be to suppress pathogenic fibrosis while preserving its neuroprotective and immunosurveillance functions.

### 4.4 Therapeutic targets related to IL-11

Interleukin-11 (IL-11), a member of the interleukin-6 (IL-6) cytokine family, was initially described for its role in platelet production. Recent studies have revealed that its critical involvement in fibrosis across multiple organ systems (59). IL-11 signals through various stromal and epithelial cells via both autocrine and paracrine mechanisms, activating multiple downstream pathways, including JAK/STAT3, ERK/p90RSK, mTOR and the E-cadherin switch, while concurrently inhibiting GSK3β activity. These signaling events drive mesenchymal gene expression and inflammatory processes across different cell types, and also promoting the q progression of fibrosis (60).

Therapeutic inhibition of IL-11 has been shown to confer significant antifibrotic effects. Given that IL-11 deficiency in healthy adults does not markedly impair host defense mechanisms against infection, targeting IL-11 represents a compelling and potentially safe strategy for antifibrotic therapy. Experimental approaches have been undertaken to explore fibrosis treatment through the development of IL-11 receptor antibodies and anti-IL-11 antibodies. Notable companies in this field include *Lassen Therapeutics*, *Boehringer Ingelheim*, and *Mabwell Therapeutics*, all of which are developing experimental drugs that have demonstrated favorable efficacy and excellent safety in bleomycin-induced fibrosis models. Among these, *Lassen Therapeutics* has developed a drug coded as NCT05331300, which has shown efficacy in treating thyroid eye disease and preventing orbital fibrosis (59, 60) (Table 2).

**Table 2:** Recent targeted therapeutic strategies in retinal fibrosis.

Experimental subject	Target	Analysis technique	Study design
Animal models/patients with fibrosis	TGF-β inhibitors (e.g., losartan, nintedanib, pirfenidone, triptolide, fenofibrate)	Molecular analysis, immunohistochemistry	Assessment of fibrosis severity and tissue changes
Cell culture/animal models	YAP/TAZ silencing or activation of Gα-DRD1	Cell migration, tissue stiffness analysis, fibrosis markers	Effects of signal pathway interventions on fibrosis
Mouse retina	γ-secretase inhibitor RO4929097 (Notch pathway inhibition)	Immunostaining, ECM protein expression analysis	Evaluation of fibrosis suppression effects
Human blood/tissues	C3 and C5 complement inhibitors	Plasma testing, immunohistochemistry	Assessment of complement levels and fibrosis association
Mouse models/Fibroblasts	IL-11 targeting Antibody or inhibitors	Molecular Analysis, histology	IL-11 role in fibrosis; Blockade reduces ECM deposition

Notably, current evidence positioning interleukin-11 (IL-11) as a novel anti-fibrotic target, through activation of JAK/STAT3, ERK/p90RSK, and mTOR signaling, originates entirely from systemic fibrosis models, including those of the lung, liver, kidney, and skin. Direct mechanistic validation in retinal tissue remains lacking. Moreover, thyroid eye disease, as an orbital fibrotic disorder, differs fundamentally from retinal fibrosis in cellular origin, microenvironment, and immune privilege. Therefore, clinical progress in thyroid eye disease (NCT05331300) should not be extrapolated as evidence of efficacy for retinal fibrosis. Although IL-11 exhibits an attractive safety profile due to its relatively low physiological redundancy, its therapeutic potential in retinal fibrosis requires independent target validation and efficacy confirmation in retina-specific disease models, such as laser-induced choroidal neovascularization and inherited retinal degeneration models. These studies will determine the functional responsiveness of resident retinal cells to IL-11 signaling and generate essential evidence for evaluating the suitability of this target for ophthalmic drug development.

## 5 Current limitations

Based on these candidate therapeutic targets, their long-term safety profiles and compatibility with current standards of care, including anti-VEGF therapy, require careful consideration. Currently, most antifibrotic strategies are extrapolated from models of systemic fibrotic disease. Although partial efficacy has been demonstrated in experimental models of retinal pathology, the long-term safety of their ocular application remains insufficiently characterized. Notably, broad inhibition of pleiotropic pathways such as TGF-β may disrupt physiological tissue repair processes and immune homeostasis (61). Regarding therapeutic integration, an ideal strategy would involve developing drugs capable of simultaneously targeting both angiogenesis and fibrogenesis, or using them in a sequential or combined regimen with anti-VEGF therapy. For example, certain agents (e.g., Fenofibrate) have demonstrated dual potential in preclinical models, suggesting the feasibility of combination therapy. Despite promising preclinical targets, clinical translation of anti-fibrotic strategies remains challenging.

In particular, although TGF-β is a central driver of fibrosis, its therapeutic inhibition in humans is limited by its pleiotropic physiological roles, underscoring the need to optimize combination strategies with anti-VEGF therapy (61). Therapeutic responses to Wnt, YAP/TAZ, or complement-targeted interventions in systemic fibrosis do not uniformly extend to the retina (62), likely due to its specialized microenvironment and cell-specific signaling context. Bridging this translational gap requires validation in retina-specific models and the advancement of targeted ocular delivery strategies.

## 6 Conclusion

Retinal fibrosis remains a major therapeutic challenge due to its multifactorial nature and the complexity of NVU-driven pathogenic pathways. This review highlights key molecular processes by which NVU-associated signaling mechanisms can be targeted. Continued advances in this field may facilitate the identification of novel therapeutic targets in retinal fibrosis, ultimately paving the way for more effective antifibrotic strategies to address unmet clinical needs.
